# Association of genetic risk and outcomes in patients with atrial fibrillation: interactions with early rhythm control in the EAST-AFNET4 trial

**DOI:** 10.1093/cvr/cvad027

**Published:** 2023-06-02

**Authors:** Shinwan Kany, Christoph Al-Taie, Carolina Roselli, James P Pirruccello, Katrin Borof, Carla Reinbold, Anna Suling, Linda Krause, Bruno Reissmann, Renate B Schnabel, Tanja Zeller, Antonia Zapf, Karl Wegscheider, Larissa Fabritz, Patrick T Ellinor, Paulus Kirchhof

**Affiliations:** Department of Cardiology, University Heart and Vascular Center Hamburg, University Medical Center Hamburg Eppendorf, Martinistraße 52, 20248 Hamburg, Germany; University Center of Cardiovascular Science, University Heart and Vascular Center Hamburg, University Medical Center Hamburg Eppendorf, Hamburg, Germany; German Center for Cardiovascular Research (DZHK), Hamburg/Kiel/Lübeck, Germany; Cardiovascular Disease Initiative, The Broad Institute of MIT and Harvard, Cambridge, MA, USA; Cardiovascular Research Center, Massachusetts General Hospital, Boston, MA, USA; Department of Cardiology, University Heart and Vascular Center Hamburg, University Medical Center Hamburg Eppendorf, Martinistraße 52, 20248 Hamburg, Germany; University Center of Cardiovascular Science, University Heart and Vascular Center Hamburg, University Medical Center Hamburg Eppendorf, Hamburg, Germany; German Center for Cardiovascular Research (DZHK), Hamburg/Kiel/Lübeck, Germany; Cardiovascular Disease Initiative, The Broad Institute of MIT and Harvard, Cambridge, MA, USA; Cardiovascular Disease Initiative, The Broad Institute of MIT and Harvard, Cambridge, MA, USA; Cardiovascular Research Center, Massachusetts General Hospital, Boston, MA, USA; Division of Cardiology, University of California San Francisco, San Francisco, CA, USA; Department of Cardiology, University Heart and Vascular Center Hamburg, University Medical Center Hamburg Eppendorf, Martinistraße 52, 20248 Hamburg, Germany; University Center of Cardiovascular Science, University Heart and Vascular Center Hamburg, University Medical Center Hamburg Eppendorf, Hamburg, Germany; Department of Cardiology, University Heart and Vascular Center Hamburg, University Medical Center Hamburg Eppendorf, Martinistraße 52, 20248 Hamburg, Germany; University Center of Cardiovascular Science, University Heart and Vascular Center Hamburg, University Medical Center Hamburg Eppendorf, Hamburg, Germany; Institute of Medical Biometry and Epidemiology, University Medical Center Hamburg Eppendorf, Hamburg, Germany; Institute of Medical Biometry and Epidemiology, University Medical Center Hamburg Eppendorf, Hamburg, Germany; Department of Cardiology, University Heart and Vascular Center Hamburg, University Medical Center Hamburg Eppendorf, Martinistraße 52, 20248 Hamburg, Germany; University Center of Cardiovascular Science, University Heart and Vascular Center Hamburg, University Medical Center Hamburg Eppendorf, Hamburg, Germany; German Center for Cardiovascular Research (DZHK), Hamburg/Kiel/Lübeck, Germany; Department of Cardiology, University Heart and Vascular Center Hamburg, University Medical Center Hamburg Eppendorf, Martinistraße 52, 20248 Hamburg, Germany; University Center of Cardiovascular Science, University Heart and Vascular Center Hamburg, University Medical Center Hamburg Eppendorf, Hamburg, Germany; German Center for Cardiovascular Research (DZHK), Hamburg/Kiel/Lübeck, Germany; Department of Cardiology, University Heart and Vascular Center Hamburg, University Medical Center Hamburg Eppendorf, Martinistraße 52, 20248 Hamburg, Germany; University Center of Cardiovascular Science, University Heart and Vascular Center Hamburg, University Medical Center Hamburg Eppendorf, Hamburg, Germany; German Center for Cardiovascular Research (DZHK), Hamburg/Kiel/Lübeck, Germany; Institute of Medical Biometry and Epidemiology, University Medical Center Hamburg Eppendorf, Hamburg, Germany; Institute of Medical Biometry and Epidemiology, University Medical Center Hamburg Eppendorf, Hamburg, Germany; Department of Cardiology, University Heart and Vascular Center Hamburg, University Medical Center Hamburg Eppendorf, Martinistraße 52, 20248 Hamburg, Germany; University Center of Cardiovascular Science, University Heart and Vascular Center Hamburg, University Medical Center Hamburg Eppendorf, Hamburg, Germany; German Center for Cardiovascular Research (DZHK), Hamburg/Kiel/Lübeck, Germany; Institute of Cardiovascular Sciences, University of Birmingham, Wolfson Drive, Birmingham, UK; Cardiovascular Disease Initiative, The Broad Institute of MIT and Harvard, Cambridge, MA, USA; Cardiovascular Research Center, Massachusetts General Hospital, Boston, MA, USA; Department of Cardiology, University Heart and Vascular Center Hamburg, University Medical Center Hamburg Eppendorf, Martinistraße 52, 20248 Hamburg, Germany; University Center of Cardiovascular Science, University Heart and Vascular Center Hamburg, University Medical Center Hamburg Eppendorf, Hamburg, Germany; German Center for Cardiovascular Research (DZHK), Hamburg/Kiel/Lübeck, Germany; Institute of Cardiovascular Sciences, University of Birmingham, Wolfson Drive, Birmingham, UK

**Keywords:** Atrial fibrillation, Polygenic risk scores, Rhythm control, Heart failure, Stroke

## Abstract

**Aims:**

The randomized Early Treatment of Atrial Fibrillation for Stroke Prevention Trial found that early rhythm control reduces cardiovascular events in patients with recently diagnosed atrial fibrillation (AF) compared with usual care. How genetic predisposition to AF and stroke interacts with early rhythm-control therapy is not known.

**Methods and results:**

Array genotyping and imputation for common genetic variants were performed. Polygenic risk scores (PRS) were calculated for AF (PRS-AF) and ischaemic stroke risk (PRS-stroke). The effects of PRS-AF and PRS-stroke on the primary outcome (composite of cardiovascular death, stroke, and hospitalization for acute coronary syndrome or worsening heart failure), its components, and recurrent AF were determined.

A total of 1567 of the 2789 trial patients were analysed [793 randomized to early rhythm control; 774 to usual care, median age 71 years (65–75), 704 (44%) women]. Baseline characteristics were similar between randomized groups. Early rhythm control reduced the primary outcome compared with usual care [HR 0.67, 95% CI: (0.53, 0.84), *P* < 0.001]. The randomized intervention, early rhythm control, did not interact with PRS-AF (interaction *P* = 0.806) or PRS-stroke (interaction *P* = 0.765). PRS-AF was associated with recurrent AF [HR 1.08 (01.0, 1.16), *P* = 0.047]. PRS-stroke showed an association with the primary outcome [HR 1.13 (1.0, 1.27), *P* = 0.048], driven by more heart failure events [HR 1.23 (1.05–1.43), *P* = 0.010] without differences in stroke [HR 1.0 (0.75, 1.34), *P* = 0.973] in this well-anticoagulated cohort. In a replication analysis, PRS-stroke was associated with incident AF [HR 1.16 (1.14, 1.67), *P* < 0.001] and with incident heart failure in the UK Biobank [HR 1.08 (1.06, 1.10), *P* < 0.001]. The association with heart failure was weakened when excluding AF patients [HR 1.03 (1.01, 1.05), *P* = 0.001].

**Conclusions:**

Early rhythm control is effective across the spectrum of genetic AF and stroke risk. The association between genetic stroke risk and heart failure calls for research to understand the interactions between polygenic risk and treatment.

**Registration:**

ISRCTN04708680, NCT01288352, EudraCT2010-021258-20, www.easttrial.org

## Graphical Abstract

**Graphical Abstract:**
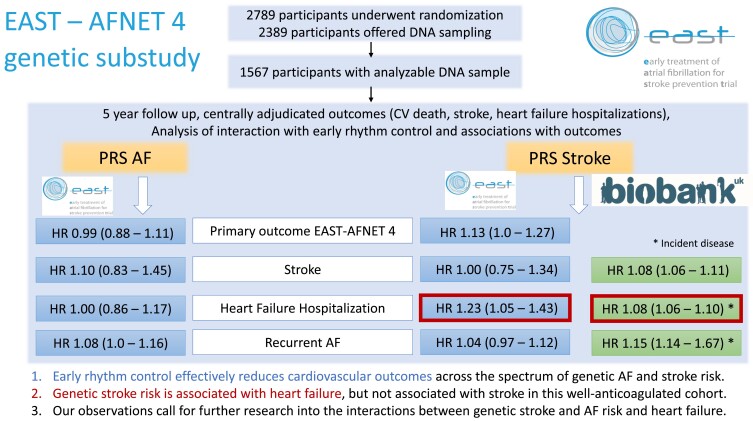

**Time of primary review: 16 days See the editorial comment for this article ‘Has genetic disposition implications for treatment decisions in atrial fibrillation?’, by F. Bourier and H. Schunkert, https://doi.org/10.1093/cvr/cvad098.**

## 1. Introduction

The Early Treatment of Atrial Fibrillation for Stroke Prevention Trial (EAST-AFNET4) showed that early rhythm control (ERC) reduces a composite of cardiovascular death, stroke, and hospitalization for worsening heart failure (HF) or acute coronary syndrome compared with usual care (UC) when added to oral anticoagulation and therapy of concomitant cardiovascular conditions.^1^ Several sub-analyses demonstrate that early rhythm control is effective independent of atrial fibrillation (AF) symptoms,^2^ HF,^3^ and AF pattern.^4^ The treatment effect appears to be mediated by sinus rhythm,^5^ suggesting that factors that render rhythm control more difficult may interact with the treatment effect of early rhythm control.

The heritability of AF has been described as high as ∼62% in twins and ∼22% in the general population.^6,7^ Polygenic risk scores (PRS) for AF using data from large genome-wide association studies (GWAS) can quantify the genetic risk for incident AF with a three- to six-fold risk difference.^8^ Additionally, studies using PRS for stroke can identify AF patients with a four-fold increase in stroke risk when otherwise classified as low risk by CHA_2_DS_2_-VASc.^9^ Similarly, integration of the genetic risk for stroke with clinical risk factors increased the risk prediction for stroke.^10^ Several observational data sets suggest that AF risk variants are associated with recurrent AF on different rhythm-control therapies.^11–13^ In coronary artery disease, high genetic risk by PRS has been shown to predict benefit from lipid-lowering therapy.^14^ A recent scientific statement document from the American Heart Association called for more research into PRS and rhythm-control therapy in patients with AF.^15^ Whether genetic risk is associated with adverse events or response to treatment in AF is not known. Here, we analysed the interaction between genetic AF and stroke risk and cardiovascular events in the EAST-AFNET4 biosample study.

## 2. Methods

### 2.1 Trial population and intervention

The EAST-AFNET4 trial was a multi-centre investigator-initiated, parallel-group, open, blinded-outcome-assessment trial. Patients with recently diagnosed AF (<1 year) and cardiovascular risk factors were randomized to early rhythm control or usual care. Inclusion criteria were either patients aged >75 years or prior stroke or two of the following criteria: age >65 years, female sex, HF, hypertension, diabetes mellitus, severe coronary artery disease, chronic kidney disease [modification of diet in renal disease stage 3 or 4 (glomerular filtration rate 15–59 mL/1.73 m^2^ of body surface area)], and left ventricular hypertrophy (diastolic septal wall width >15 mm). In the main trial, 2789 patients across 135 sites were 1:1 randomized to either ERC (*n* = 1395) or UC (*n* = 1394).^1^ Patients randomized to ERC received anti-arrhythmic drugs, catheter ablation, or cardioversion directly after randomization.

Rhythm was assessed for all patients at 1 year and 2 years of follow-up. Additionally, recurrent AF was defined in the study protocol as any symptomatic or asymptomatic AF episode (clinically lasting longer than 30 s) after successful index therapy that is documented in an electrocardiogram (ECG). When AF was only documented by a single telemetric ECG, verification of the presence of AF by another technique (standard ECG, Holter ECG or implanted ECG) was required. Any documentation of AF in a standard ECG or Holter ECG constituted an AF recurrence.

Patients in the ERC group were also given a single-lead ECG (Vitaphone) to transmit ECGs twice per week and when symptomatic. Documentation of recurrent AF triggered an escalation of rhythm-control therapy as clinically indicated. In UC, rate control was the initial strategy and rhythm control was only used when AF-related symptoms persisted on optimal rate control.

### 2.2 Biosample sub-study

The EAST-AFNET4 biosample study was started a few months after the initiation of the trial. Participation was offered to 2390/2789 patients. These patients were asked to donate a blood sample at baseline, documented by a separate informed consent form. 1600/2390 patients (67%) consented to blood sampling and analysis. Samples were shipped to the central processing and storage facility at UKE Hamburg (Hamburg, Germany), spun, and frozen for later analysis. DNA was isolated from buffy coat prepared from EDTA blood samples.^16^ DNA samples were shipped to the Broad Institute (Cambridge, USA). Thirty-three patient samples did not pass quality control for genotyping. Samples were genotyped on the Infinium PsychArray-24 v1.2 BeadChip and called with GenomeStudio. The pre-imputation quality control included sample level filtering (call rate < 98%, excess heterozygosity > ±0.2) and variant level filtering (call rate < 98%, Hardy-Weinberg Equilibrium *P*-value < 1 × 10^–8^). Imputation was performed on the TOPMed imputation server with the TOPMed Freeze 5 dataset as reference.^17,18^ Polygenic risk scores (PRS) for incident AF risk (PRS-AF) and ischaemic stroke risk (PRS-stroke, O’Sullivan *et al*.^10^) were calculated using PLINK2. PRS-AF was constructed by Khera *et al*. using the LDPred method (best performing PRS) and a discovery GWAS of 17 931 cases and 115 142 controls.^8^ PRS-stroke was constructed by O’Sullivan *et al*. using lassosum and a discovery GWAS of 7193 cases and 204 570 controls.^10^ EAST-AFNET 4 was not used for the derivation of the PRS-weights. Sum scores were obtained and PRS calculated based on TOPMed imputed genotype dosages with an imputation quality measure for each variant >0.3 and then summed across the genome. After quality control and imputation, we used 6 363 335 single nucleotide variants (out of 6 730 541) to calculate PRS-AF and 516 013 single nucleotide variants (out of 530 933) to calculate the PRS-stroke.

### 2.3 UK Biobank analysis

The UK Biobank is a prospective cohort study of over 500 000 participants from the United Kingdom. Patients between 40 and 69 years of age were recruited from 2006 until 2010^19^ including comprehensive biosamples including DNA.^20^ Health-related outcome data are available via self-reports and death registries as well as Hospital Episode Statistics. The PRS-stroke was applied to all individuals in the UK Biobank who were centrally adjudicated to be in an ancestrally relatively homogenous group termed ‘white British’ by the UK Biobank and who were free of the diseases of interest at baseline. The PRS-stroke score was tested for association with incident AF, HF, and stroke using Cox proportional hazards models that were adjusted for sex, the first five principal components of ancestry, the genotyping array, and the cubic splines of age at UK Biobank enrolment, height, weight, body mass index, systolic blood pressure, and diastolic blood pressure. Hazard ratios are reported as a 1 SD change of the PRS on the risk of incident disease.

### 2.4 Statistical analysis

Descriptive statistics present mean and standard deviation or median and interquartile range (IQR) for metric variables and frequencies and percentages for categorical variables. We visually checked if the continuous genetic risk was approximately normally and equally distributed and the categorized genetic risk was equally distributed across treatment groups. For visualization of time-to-event endpoints, we used Aalen Johansen cumulative incidence estimators to account for the competing risk of all-cause death.

PRS was assessed as a standardized continuous variable, first. Additionally, patients were grouped into low (quintile one), intermediate (quintile two to four), and high genetic risk (quintile five) for PRS-AF_categorized_ and PRS-stroke_categorized_.^14^ The following outcomes were analysed by PRS score (standardized continuous and categorical) for AF risk and ischaemic stroke risk separately: time to the primary outcome of the trial, a composite of cardiovascular death, stroke (ischaemic or haemorrhagic), hospitalization for acute coronary syndrome or worsening of HF and its components; and time to recurrent AF. To identify a possible interaction between the treatment group and genetic risk, we calculated Cox regression models, with an interaction term between treatment group and the categorical genetic risk score. To obtain hazard ratios (HRs) for the treatment effect for each level (low, intermediate, and high) of categorized genetic risk scores, we followed the approach suggested by Figueiras *et al*., i.e. we calculated three models with differing genetic risk levels as reference and interaction terms for treatment group and genetic risk.^21^ Then, we compared each of these models with analysis of variance against a nested version without interaction terms to receive a *P*-value for the overall interaction.

The interaction term was removed when the interaction *P*-value was >0.05. To calculate HRs for the treatment effect adjusted for genetic risk, we used Cox regression models with continuous genetic risk as an adjusting variable. A frailty term for recruitment centre ID was included in all Cox regression models. We present the treatment effects as hazard ratios with 95% confidence intervals. Due to the explorative design of the study, no adjustment for multiple testing was done, that is, *P-*values are descriptive. For all analyses, we used Stata software (StataCorp), version 16.1, R version 4.2.1, and Python version 3.8.13.

## 3. Results

### 3.1 Patient characteristics by randomized group and genetic risk category

**Figure 1:**
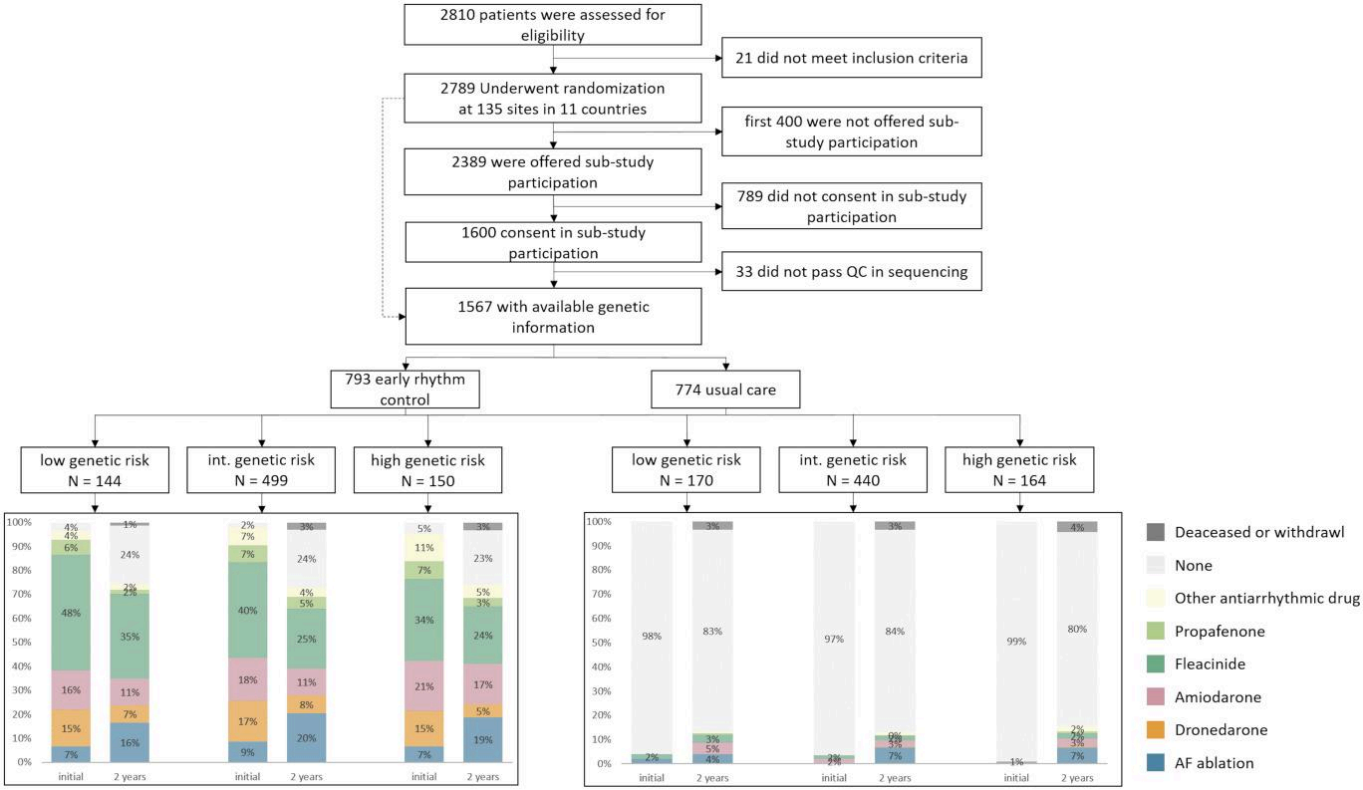
Consort flow chart of the patients included in the analysis for genetic AF risk. Treatment is shown split by randomized group and by risk groups of PRS AF.

**Figure 2:**
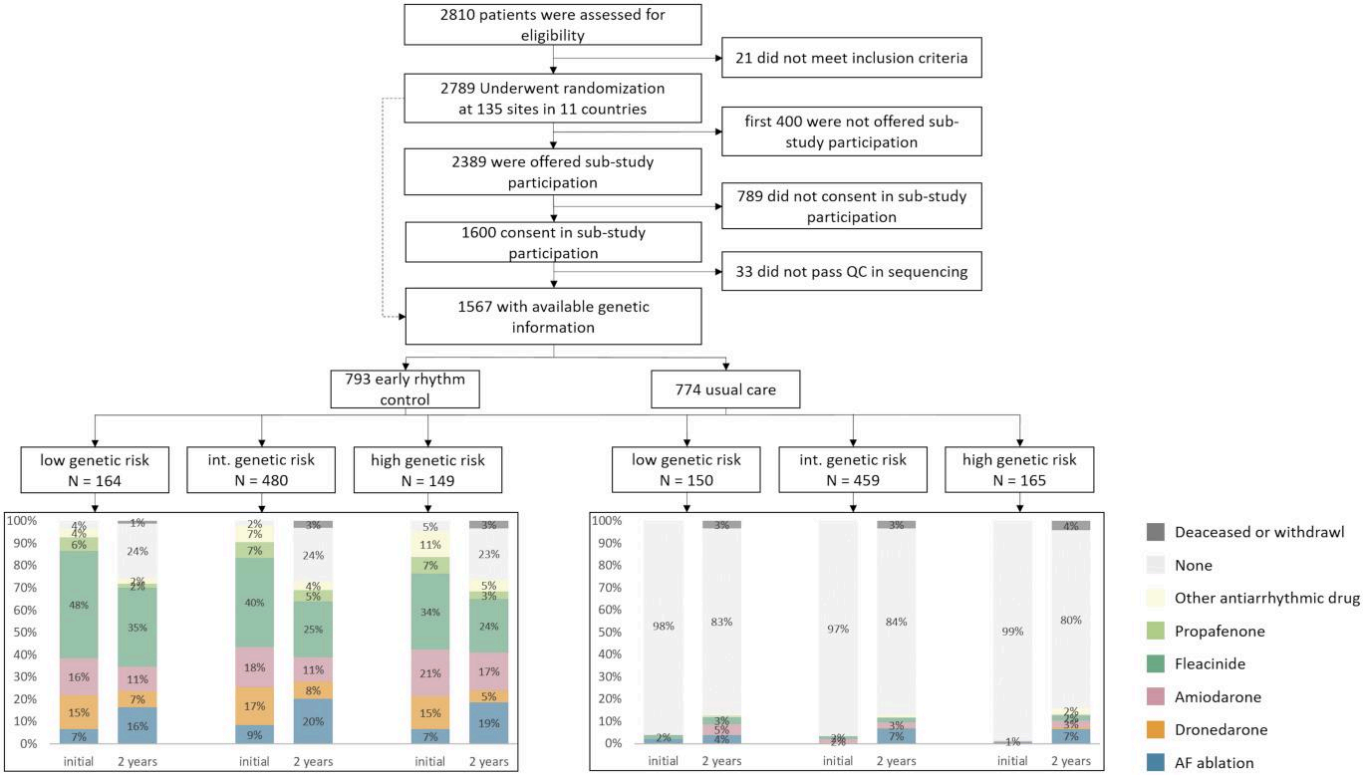
Consort flow chart of the patients included in this analysis for genetic stroke risk. Treatment is shown split by randomized group and by risk groups of PRS-stroke.

After genotyping, imputation, and stringent quality control, 1567 patients were available for the PRS analyses (*Figures 1* and *2*). Of these, 793 were randomized to ERC and 774 to UC (*Table 1*). Baseline characteristics were similar between randomized groups including median age [ERC 71.0 (65.0, 75.0) years, UC 71.0 (66.0, 76.0) years], female sex (ERC 352/793; UC 352/774), and mean CHA_2_DS_2_-VASc score (*Table 1*). Similar to the entire trial cohort,^22^ more than 90% of patients in both randomized groups received anticoagulation and had well-controlled concomitant cardiovascular conditions (e.g. blood pressure, *Table 1*).

**Table 1:** Baseline characteristics of patients in EAST-AFNET4 biosample sub-study receiving either early rhythm control or usual care

		Treatment group		
		Early rhythm control (*N* = 793)	Usual care (*N* = 774)	Total (*N* = 1567)
Age	Median (IQR)	71.0 (65.0;75.0)	71.0 (66.0;76.0)	71.0 (66.0;76.0)
Gender (female)		352 (44.4%)	352 (45.5%)	704 (44.9%)
PRS-AF score	Low	144 (18.2%)	170 (22.0%)	314 (20.0%)
	Intermediate	499 (62.9%)	440 (56.8%)	939 (59.9%)
	High	150 (18.9%)	164 (21.2%)	314 (20.0%)
PRS-stroke score	Low	164 (20.7%)	150 (19.4%)	314 (20.0%)
	Intermediate	480 (60.5%)	459 (59.3%)	939 (59.9%)
	High	149 (18.8%)	165 (21.3%)	314 (20.0%)
Body mass index (kg/m²) (*N* = 1560)	Mean ± SD	29.4 ± 5.4	29.5 ± 5.3	29.4 ± 5.3
Type of AF	First episode	289 (36.4%)	266 (34.4%)	555 (35.4%)
	Paroxysmal	297 (37.5%)	285 (36.8%)	582 (37.1%)
	Persistent	207 (26.1%)	223 (28.8%)	430 (27.4%)
Heart rhythm (Sinus rhythm at baseline)		446 (56.2%)	431 (55.7%)	877 (56.0%)
Days since AF diagnosis	Mean ± SD	80.2 ± 183.3	82.7 ± 164.0	81.4 ± 174.0
Previous cardioversion		291/776 (37.5%)	272/773 (35.2%)	563/1549 (36.3%)
Prior stroke or transient ischaemic attack		114 (14.4%)	81 (10.5%)	195 (12.4%)
**Concomitant conditions**				
At least mild cognitive impairment (MoCA < 26)		333/771 (43.2%)	329/755 (43.6%)	662/1526 (43.4%)
Arterial hypertension		704 (88.8%)	681 (88.0%)	1385 (88.4%)
Systolic blood pressure (mmHg) (*N* = 1562)	Mean ± SD	136.9 ± 19.6	137.3 ± 19.3	137.1 ± 19.4
Diastolic blood pressure (mmHg) (*N* = 1562)	Mean ± SD	81.3 ± 12.1	81.5 ± 12.0	81.4 ± 12.0
Stable HF		232 (29.3%)	239 (30.9%)	471 (30.1%)
CHA2DS2-Vasc score	Mean ± SD	3.4 ± 1.3	3.3 ± 1.3	3.3 ± 1.3
	Median (IQR)	3.0 (2.0;4.0)	3.0 (2.0;4.0)	3.0 (2.0;4.0)
Valvular heart disease		324/793 (40.9%)	316/773 (40.9%)	640/1566 (40.9%)
Chronic kidney disease (MDRD stage III or IV)		99 (12.5%)	94 (12.1%)	193 (12.3%)
**Medication at discharge**				
Oral anticoagulation (NOAC and VKA) at discharge		737 (92.9%)	703 (90.8%)	1440 (91.9%)
Digoxin or digitoxin at discharge		27 (3.4%)	47 (6.1%)	74 (4.7%)
Beta blockers at discharge		584 (73.6%)	656 (84.8%)	1240 (79.1%)
ACE inhibitors or angiotensin II receptor blocker at discharge		545 (68.7%)	548 (70.8%)	1093 (69.8%)
Mineralocorticoid receptor antagonist at discharge		56 (7.1%)	45 (5.8%)	101 (6.4%)
Diuretics at discharge		300 (37.8%)	306 (39.5%)	606 (38.7%)
Statin at discharge		373 (47.0%)	319 (41.2%)	692 (44.2%)
Inhibitor of platelet aggregation at discharge		102 (12.9%)	116 (15.0%)	218 (13.9%)
Rhythm control at baseline	Ablation	56 (7.1%)	0 (0.0%)	56 (3.6%)
	AAD	714 (90.0%)	21 (2.7%)	735 (46.9%)
	None	23 (2.9%)	753 (97.3%)	776 (49.5%)

Presented as mean ± standard deviation, Median and Interquartile range (IQR) or total numbers and percentages in brackets.

PRS, polygenic risk score; AF, atrial fibrillation; MoCA, montreal cognitive assessment; MDRD, modification of diet in renal disease; NOAC, non-VKA oral anticoagulant; VKA, vitamin K antagonist; AAD, anti-arrhythmic drug.

The distribution of genetic risk followed a normal distribution for each PRS per treatment group (see Supplementary material online, Figure S1). In the three risk groups of PRS-AF_categorized,_ median age (low risk: 72.0 (67.0, 76.0) years, intermediate risk: 71.0 (66.0, 75.0) years, high risk: 70.0 (65.0, 75.0) years) and sex (low risk: 43.9% female, intermediate risk: 45.7%, high risk: 43.6%) were similarly distributed (see Supplementary material online, *Table S1*). Patients with a high genetic risk for AF were younger, had less previous cardioversion and more HF (see Supplementary material online, *Table S1*).

The same was found in the PRS-stroke_categorized_ groups, median age (low risk: 71.0 years, intermediate risk: 71.0 years, high risk: 70.0 years), and sex (low risk: 46.2% female, intermediate risk: 43.7%, high risk: 47.5%; see Supplementary material online, *Table S2*).

### 3.2 Early rhythm control is effective and safe across the spectrum of genetic AF and stroke risk

Consistent with the main trial,^1^ ERC reduced the primary outcome compared to usual care in the biosample sub-population (HR 0.67, 95% CI: 0.53–0.84, *P* < 0.001, *Table 2a*). ERC was associated with reduced hospitalizations for acute coronary syndrome (HR 0.52, 95% CI: 0.30–0.89, *P* = 0.019), reduced cardiovascular deaths (HR 0.62, 95% CI: 0.40–0.95, *P* = 0.029), and reduced strokes (HR 0.52, 95% CI: 0.29–0.94, *P* = 0.03). ERC also reduced recurrent AF (HR 0.76, 95% CI: 0.65–0.88, *P* < 0.001) compared to UC. The genetic risk for AF did not interact with the treatment effect on the primary composite outcome (interaction *P* = 0.806) nor its components or recurrent AF (see Supplementary material online, *Table S3*). Similarly, the PRS-stroke score and treatment group did not interact with the primary composite outcome (interaction *P* = 0.765) (see Supplementary material online, *Table S4*, event rates by genetic risk category given in Supplementary material online, *Tables S5* and *S6*).

**Table 2a:** Treatment effect on outcomes in the EAST-AFNET 4 trial ‘PRS’-sub-population adjusted for genetic AF risk

Outcome	Early rhythm control	Usual care	Treatment effect^a^	*P*-value
Primary outcome—events/person-year (incidence/100 person-years)	125/3810.0 (3.3)	171/3729.0 (4.6)	0.67 (0.53, 0.84)	<0.001
Components of primary outcome—events/person-year (incidence/100 person-years)				
Death from cardiovascular causes	35/4002.0 (0.9)	54/3845.0 (1.4)	0.62 (0.40, 0.95)	0.029
Stroke	18/3955.0 (0.5)	33/3769.0 (0.9)	0.52 (0.29, 0.94)	0.03
Hospitalization with worsening of HF	74/3851.0 (1.9)	90/3618.0 (2.5)	0.77 (0.56, 1.04)	0.094
Hospitalization with acute coronary syndrome	21/3941.0 (0.5)	38/3750.0 (1.0)	0.52 (0.30, 0.89)	0.019
Secondary outcome— events/person-year (incidence/100 person-years)				
Recurrent atrial fibrillation	322/2620.0 (12.3)	391/2204.0 (17.7)	0.76 (0.65, 0.88)	<0.001

Number of events per person years (incidence per 100 person-years) given.

expressed as HR from Cox regression model adjusted for continuous genetic AF risk and centre as shared frailty term.

### 3.3 Associations of genetic AF and stroke risk with trial outcomes

**Figure 3:**
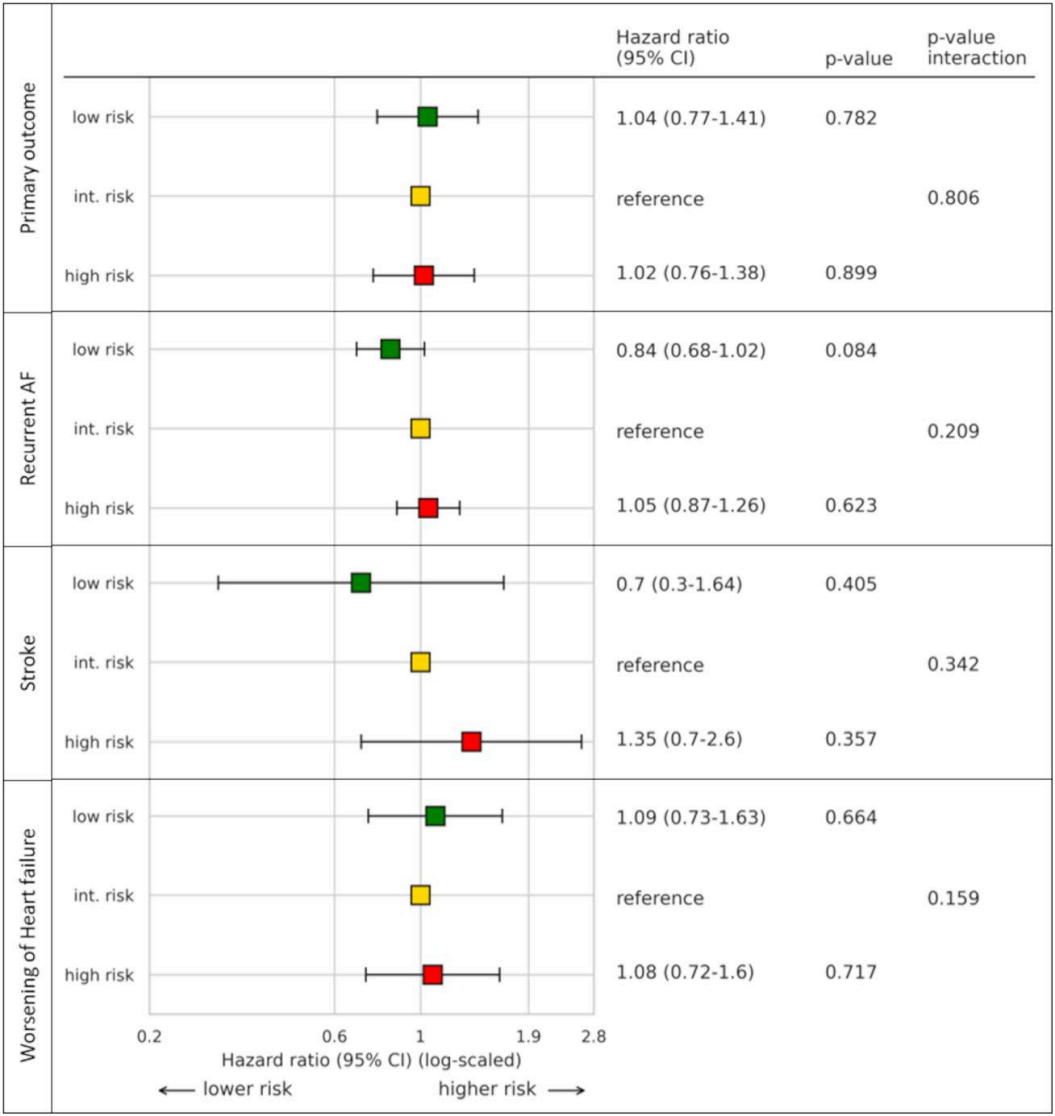
Association of AF genetic risk (PRS-AF) categories classified by quintile and occurrence of the primary composite endpoint, occurrence of recurrent AF, occurrence of stroke and hospitalization for worsening of HF. Hazard ratios and *P*-values resulting from Cox proportional hazards models with categorized PRS-AF as independent variable interacting with treatment group and a shared frailty term for centre. Int. risk, intermediate risk; AF, atrial fibrillation.

The continuous genetic risk for AF was not associated with the primary composite outcome (cardiovascular death, stroke, hospitalization for worsening of HF, and acute coronary syndrome) (HR 0.99, 95% CI: 0.88–1.11, *P* = 0.867) (*Table 2b, Figure S2*). The PRS-AF score was associated with recurrent AF with a moderate effect size (HR 1.08, 95% CI: 1.0–1.16, *P* = 0.047). When looking at genetic risk as categorical values, a trend toward lower risk for recurrent AF in patients with low PRS-AF_categorized_ risk was observed compared to patients with an intermediate risk (HR 0.84, 95% CI: 0.68–1.02, *P* = 0.084, *Figure 3*).

**Table 2b:** Association of PRS-AF as a continuous variable with cardiovascular outcomes in the EAST-AFNET 4 trial

Genetic AF risk		
Outcome	Hazard ratio (95% CI)	*P*-value
EAST-AFNET 4 primary outcome	0.99 (0.88–1.11)	0.867
Components primary outcome		
Death from cardiovascular causes	0.99 (0.80–1.23)	0.962
Stroke	1.10 (0.83–1.45)	0.506
Worsening of HF	1.00 (0.86–1.17)	0.974
Acute coronary syndrome	0.91 (0.70–1.19)	0.492
Secondary outcome		
Recurrent atrial fibrillation	1.08 (1.0–1.16)	0.047

**Figure 4:**
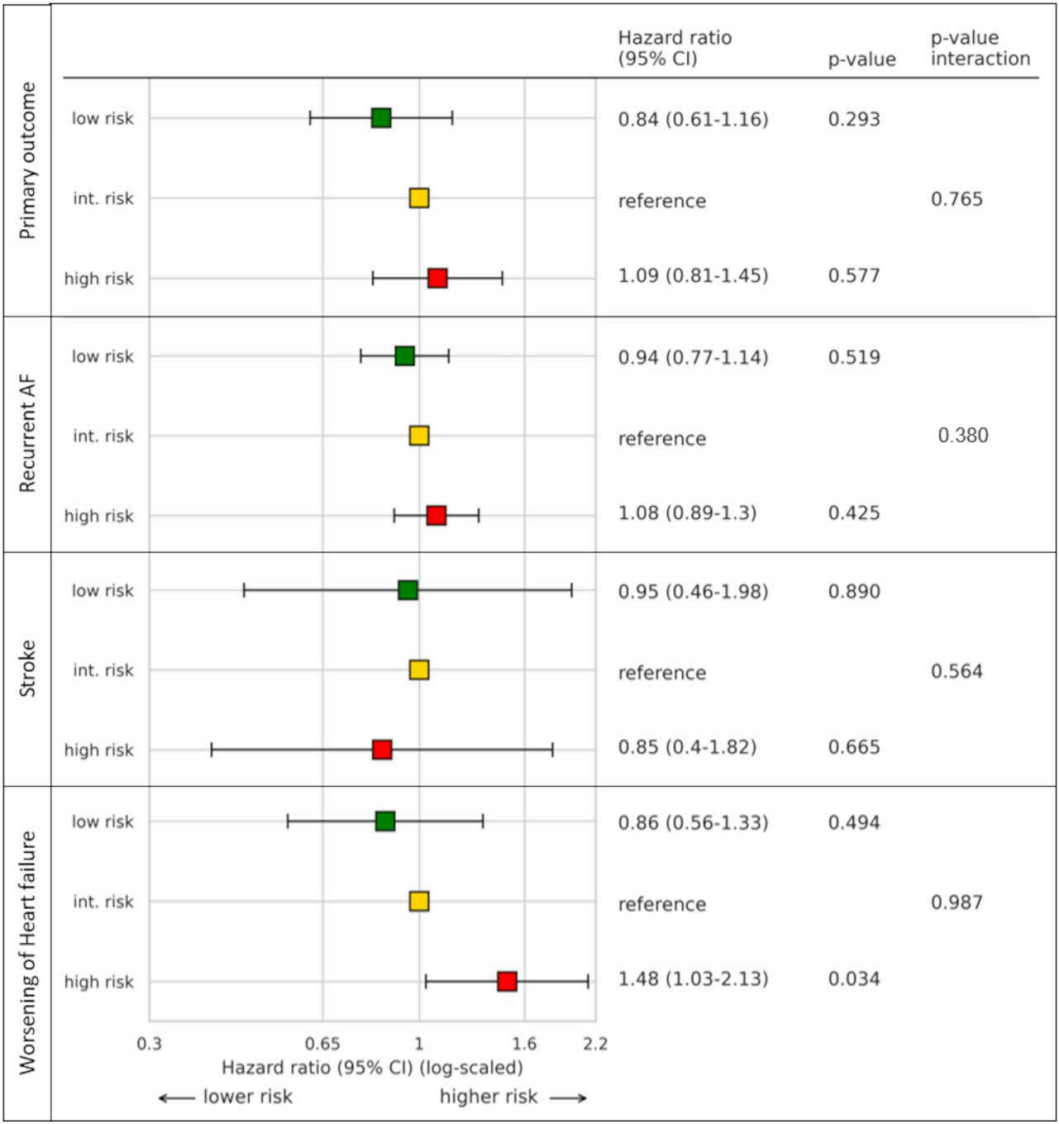
Association of genetic risk for ischaemic stroke (PRS-stroke) categories classified by quintile and occurrence of the primary composite endpoint, occurrence of recurrent AF, occurrence of stroke, and hospitalization for worsening of HF. Hazard ratios and *P*-values resulting from Cox proportional hazards models with categorized PRS-stroke as independent variable interacting with treatment group and a shared frailty term for centre. Int. risk, intermediate risk; AF, atrial fibrillation.

The continuous genetic risk for stroke showed an association with the primary composite outcome (HR 1.13, 95% CI: 1.0–1.27, *P* = 0.048) but was not associated with stroke (ischaemic or heamorrhagic, HR 1.00, 95% CI: 0.75–1.34, *P* = 0.973, *Figure S2*) in this well-anticoagulated population (*Table 2c*, > 90% on adequate anticoagulation). The genetic stroke risk was associated with worsening of HF (HR 1.23, 95% CI: 1.05–1.43, *P* = 0.010) but not with recurrent AF (HR 1.04, 95% CI: 0.97–1.12, *P* = 0.228). In the categorized analysis, high PRS-stroke_categorized_ risk was associated with an increased risk of hospitalization for worsening HF in comparison to intermediate risk (HR 1.48, 95% CI: 1.03–2.13, *P* = 0.034, *Figure 4*).

**Table 2c:** Association of PRS-stroke as a continuous variable with cardiovascular outcomes in the EAST-AFNET 4 trial

Genetic stroke risk		
Outcome	Hazard ratio (95% CI)	*P*-value
EAST-AFNET 4 primary outcome	1.13 (1.0–1.27)	0.048
Components of primary outcome		
Death from cardiovascular causes	1.14 (0.92–1.40)	0.237
Stroke	1.00 (0.75–1.34)	0.973
Worsening of HF	1.23 (1.05–1.43)	0.010
Acute coronary syndrome	0.94 (0.72–1.23)	0.670
Secondary outcome		
Recurrent atrial fibrillation	1.04 (0.97–1.12)	0.228

Hazard ratio as 1 SD change of the PRS on the risk of an outcome, calculated using Cox regression models adjusted for treatment group and with centre as a shared frailty term.

To further investigate this finding, we applied the PRS-stroke to up to 407 311 UKB participants, where we found an association of the risk score with incident HF (*N* = 12 388 cases; HR 1.08, 95% CI: 1.06–1.10, *P* < 0.001, *Table 3*). This association was weaker when excluding participants in the UKB who first developed AF (N = 8713 cases without AF before HF; HR 1.03, 95% CI: 1.01–1.05, *P* = 0.001). The PRS-stroke showed a robust association with AF risk (N = 24 767 cases; HR 1.15, 95% CI: 1.14–1.67, *P* < 0.001) in the UKB, in addition to the expected association with ischaemic stroke (N = 3490 cases; HR 1.11, 95 CI: 1.08–1.14, *P* < 0.001).

**Table 3:** Association of PRS-stroke with incident disease in the UK biobank using adjusted cox regression models. Hazard ratio as 1 SD change of the PRS on the risk of incident disease

Disease	*N* overall cohort	*N* incident disease	Hazard ratio (95% CI)	*P*-value
HF	406 710	12 388	1.08 (1.06–1.10)	<0.001
HF (excluding AF)	401 999	8713	1.03 (1.01–1.05)	0.001
Atrial fibrillation/atrial flutter (AF)	403 192	24 767	1.15 (1.14–1.67)	<0.001
Stroke	402 532	4468	1.08 (1.06–1.11)	<0.001
Ischemic stroke	407 311	3490	1.11 (1.08–1.14)	<0.001

AF, atrial fibrillation.

### 3.4 Safety events

Safety events were rare and not different across the PRS-AF and PRS-stroke risk categories, including events related to anti-arrhythmic drug therapy like non-fatal cardiac arrest and drug-induced bradycardia (*Tables 4a* and 4*b*).

**Table 4a:** Safety events by genetic AF risk category and treatment group

	Genetic AF risk					
	Low genetic AF risk		Intermediate genetic AF risk		High genetic AF risk	
	Early rhythm control	Usual care	Early rhythm control	Usual care	Early rhythm control	Usual care
*n*	144	170	499	440	150	164
EAST primary composite safety outcome	18 (12.5)	32 (18.82)	79 (15.83)	65 (14.77)	26 (17.33)	27 (16.46)
Stroke	1 (0.69)	6 (3.53)	11 (2.2)	19 (4.32)	6 (4.0)	8 (4.88)
Death	5 (3.47)	14 (8.24)	24 (4.81)	28 (6.36)	6 (4.0)	12 (7.32)
Serious adverse event related to anti-arrhythmic drug therapy						
Non-fatal cardiac arrest	1 (0.69)	0 (0)	0 (0)	1 (0.23)	0 (0)	0 (0)
Drug-induced bradycardia	2 (1.39)	1 (0.59)	6 (1.2)	2 (0.45)	2 (1.33)	1 (0.61)
Torsade de Pointes tachycardia	1 (0.69)	0 (0)	0 (0)	0 (0)	0 (0)	0 (0)
Drug toxicity of AF-related drug therapy	0 (0)	1 (0.59)	5 (1.0)	0 (0)	2 (1.33)	2 (1.22)
Atrioventricular block	0 (0)	0 (0)	2 (0.4)	0 (0)	0 (0)	0 (0)
Serious adverse event related to AF ablation						
Major bleeding related to AF ablation	0 (0)	0 (0)	0 (0)	0 (0)	1 (0.67)	0 (0)
Nonmajor bleeding related to AF ablation	0 (0)	0 (0)	0 (0)	1 (0.23)	0 (0)	0 (0)
Other serious adverse event of special interest related to rhythm-control therapy						
Other event	1 (0.69)	0 (0)	0 (0)	1 (0.23)	0 (0)	0 (0)
Other cardiovascular event	0 (0)	0 (0)	2 (0.4)	0 (0)	1 (0.67)	0 (0)
Hospitalisation for AF	0 (0)	1 (0.59)	7 (1.4)	0 (0)	1 (0.67)	0 (0)
Syncope	0 (0)	0 (0)	2 (0.4)	0 (0)	1 (0.67)	1 (0.61)
Hospitalization for worsening of HF with decompensated HF	1 (0.69)	0 (0)	0 (0)	0 (0)	1 (0.67)	0 (0)
Implantation of a pacemaker, ICD, CRT or any other	1 (0.69)	0 (0)	3 (0.6)	1 (0.23)	1 (0.67)	1 (0.61)

AF, atrial fibrillation; HF, heart failure; ICD, implantable cardiac device; CRT, cardiac resynchronization therapy.

**Table 4b:** Safety events by genetic stroke risk category and treatment group

	Genetic stroke risk					
	Low genetic stroke risk		Intermediate genetic stroke risk		High genetic stroke risk	
	Early rhythm control	Usual Care	Early rhythm control	Usual care	Early rhythm control	Usual care
*n*	164	150	480	459	149	165
EAST primary composite safety outcome	17 (10.37)	25 (16.67)	80 (16.67)	69 (15.03)	26 (17.45)	30 (18.18)
Stroke	5 (3.05)	5 (3.33)	9 (1.88)	23 (5.01)	4 (2.68)	5 (3.03)
Death	6 (3.66)	7 (4.67)	24 (5.0)	35 (7.63)	5 (3.36)	12 (7.27)
Serious adverse event related to anti-arrhythmic drug therapy						
Non-fatal cardiac arrest	0 (0)	0 (0)	0 (0)	0 (0)	1 (0.67)	1 (0.61)
Drug-induced bradycardia	2 (1.22)	1 (0.67)	7 (1.46)	2 (0.44)	1 (0.67)	1 (0.61)
Torsade de Pointes tachycardia	0 (0)	0 (0)	1 (0.21)	0 (0)	0 (0)	0 (0)
Drug toxicity of AF-related drug therapy	1 (0.61)	1 (0.67)	1 (0.21)	1 (0.22)	5 (3.36)	1 (0.61)
Artrioventricular block	0 (0)	0 (0)	2 (0.42)	0 (0)	0 (0)	0 (0)
Serious adverse event related to AF ablation						
Major bleeding related to AF ablation	0 (0)	0 (0)	1 (0.21)	0 (0)	0 (0)	0 (0)
Nonmajor bleeding related to AF ablation	0 (0)	1 (0.67)	0 (0)	0 (0)	0 (0)	0 (0)
Other serious adverse event of special interest related to rhythm-control therapy						
Other event	0 (0)	0 (0)	1 (0.21)	0 (0)	0 (0)	1 (0.61)
Other cardiovascular event	0 (0)	0 (0)	3 (0.62)	0 (0)	0 (0)	0 (0)
Hospitalization for AF	0 (0)	1 (0.67)	6 (1.25)	0 (0)	2 (1.34)	0 (0)
Syncope	1 (0.61)	1 (0.67)	1 (0.21)	0 (0)	1 (0.67)	0 (0)
Hospitalization for worsening of HF with decompensated HF	0 (0)	0 (0)	2 (0.42)	0 (0)	0 (0)	0 (0)
Implantation of a pacemaker, ICD, CRT or any other	1 (0.61)	1 (0.67)	4 (0.83)	1 (0.22)	0 (0)	0 (0)

AF, atrial fibrillation; HF, heart failure; ICD, implantable cardiac device; CRT, cardiac resynchronization therapy.

## 4. Discussion

### 4.1 Main findings

Early rhythm-control therapy reduces cardiovascular events in patients with AF across the spectrum of genetic risk for AF or stroke. As expected, the PRS-AF was associated with an increased risk for recurrent AF, but the attributable risk was modest (HR 1.08) reflecting the effectiveness of early rhythm-control therapy across the spectrum of genetic AF risk. Unexpectedly, PRS-stroke was associated with HF hospitalizations but not with stroke in this well-anticoagulated cohort. The association of the PRS-stroke with HF hospitalization was validated in the UK Biobank. This study shows that early rhythm control is effective cross the spectrum of genetic AF and stroke risk and that comprehensive AF therapy including anticoagulation on >90% of the patients and therapy of concomitant cardiovascular conditions reduces the otherwise observed association of PRS-stroke with stroke.

One of the goals of genomics in medicine is to tailor therapies to an individual patient to maximize efficacy while maintaining safety.^23^ Pathophysiological consideration and prior observations suggest that a higher genetic AF risk would make rhythm-control therapy more difficult, leading potentially to a dampened treatment effect or an increase in adverse events of early rhythm control in patients with a high genetic AF risk.^11,24,25^ While limited power to detect differences in events is a caveat, this analysis found that early rhythm control is effective for patients across the spectrum of AF PRS risk scores. This is most likely due to the good effectiveness and safety of modern rhythm-control therapy as applied in the EAST-AFNET 4 trial, including in patients with multiple comorbidities,^1,26^ that has been replicated in other, recent rhythm control trials.^27,28^ While we did not find an interaction of genetic risk with adverse events of rhythm-control therapy, larger genetic studies are needed to detect interactions between genetic risk and rare adverse events in AF treatment.

Using this data set from a randomized trial with capture of adjudicated events over a mean follow-up of over 5 years provides insight into the role PRS could have in AF care. While polygenic risk is thought to provide an additional layer of risk information that can be independent of clinical risk factors, the use in patients who have disease to inform treatment strategies is less well understood.^15^ A recent large analysis demonstrated that the genetic risk scores that were developed in large, homogeneous, but not very deeply phenotyped populations predict incident AF in patients with cardiovascular diseases.^29^

Our findings are in accordance with earlier reports showing an association between the first genetic risk variant on chromosome 4q25, the locus that remains prominent in current GWAS for incident AF risk, with recurrent AF on different rhythm control interventions.^11,24,30^ In contrast with this, a meta-analysis by Shoemaker *et al*. tested whether a PRS for AF is associated with recurrent AF after catheter ablation. They found high genetic risk for AF to be associated with younger age and fewer risk factors but not with AF recurrence.^31^ There are several factors that explain the divergence with our findings. For one, the Shoemaker study was a meta-analysis of 10 centres that had heterogenous patient cohorts and catheter ablation protocols. Secondly, EAST-AFNET4 enrolled patients with a mean time of AF diagnosis of around 81 days where AF-related atrial remodelling might be less important for AF recurrence than in patients with longer AF durations. Finally, this study used a different method to estimate the genetic AF risk (LDPred in the Khera score and Pruning + Thresholding in the Shoemaker study).^31^

Clinical risk factors for AF such as obesity, alcohol, or hypertension not only enable risk assessment but also provide an actionable target to modify the risk of events such as stroke whereas PRS are not modifiable.^32^ Additionally, PRS have been used to identify subgroups in clinical trials that benefit from treatments more than others. For instance, a sub-study of the FOURIER trial showed that patients without clinical risk factors or high genetic risk (highest quintile of PRS) did not benefit from evolocumab in the reduction of vascular or coronary events.^14^ In addition, a meta-analysis of three trials for primary prevention of coronary heart disease, those with a high genetic risk had the greatest relative risk reduction (RRR 44% vs. 24%).^33^ However, evidence of a role of PRS in treatment guidance in other cardiovascular diseases than coronary artery disease is sparse which is acknowledged in the recent AHA scientific statement on PRS.^15^ This study is providing important evidence to fill that gap for AF, illustrating that early rhythm-control therapy is effective and safe across the spectrum of genetic AF and stroke risk and highlighting that treatment factors, possibly including anticoagulation, modify the genetic risk for stroke.

In the present data set containing well-anticoagulated patients with AF, this PRS-stroke was not predictive of stroke but the genetic risk was associated with an increased risk of HF hospitalization. In the MEGASTROKE GWAS on which the PRS-stroke is based, the association of cardioembolic stroke with known loci for incident AF risk may provide an explanation for this observation and hint to not-anticoagulated AF as a confounding factor. The lack of association of the PRS-stroke with stroke in the EAST-AFNET 4 data set could be due to low power (51 stroke events in this sub-sample of EAST-AFNET 4). Alternatively, it could also be a consequence of the high anticoagulation rate in the trial (>90%) which is much higher than in the observed in the UK Biobank (∼30%).^34^

Our analysis in the UKB confirms the finding in the EAST-AFNET4 data set that the PRS-stroke score is associated with HF events. Variants on the *PITX2* locus were also reported in GWAS meta-analysis of HF highlighting the link of AF and HF.^35^ If AF is the mediator of the observed increased risk for HF with genetic stroke risk, as suggested by partially shared genetic architecture (*PITX2*) across all three conditions, the observed HF events might be tachymyopathy-driven events. The association was weakened when patients with incident AF were excluded, suggesting that the association is at least partially mediated by patients with AF and HF. However, limiting this hypothesis is the fact that we did not observe an increased risk of HF with genetic AF risk. Follow-up studies to elucidate our understanding of our observation may include functional genomics to study how stroke genes could lead to HF and the pathways associated with it. Furthermore, other genetic instruments such as rare-variant-burden testing in patients who have strokes and HF might help prioritize genes of interest. Whole-exome data on a large scale are available via the UKB and the All-of-Us programme.^20,36^

These hypothesis-generating findings call for further research characterizing the interactions between genetic risk for stroke and AF and cardiovascular therapies. Our data highlight that it may be premature to use PRS as selection or inclusion criteria in prospective trials as recently suggested^37^ without a deeper understanding of the interaction between PRS, cardiovascular conditions, and their treatment.

### 4.2 Strengths and limitations

Collection of analysable biosamples in a large sub-population (69%) of eligible patients randomized in the EAST-AFNET 4 trial and systematic collection of adjudicated outcomes over a 5.1-year follow-up time are strengths of this analysis. Another strength is the continuous delivery of therapy of AF including anticoagulation and treatment of cardiovascular conditions. Both genotyping and imputation for all participants were carried out in the same sequencing centre to avoid systematic bias. Although the sample size contains PRS and outcomes over a mean follow-up duration of 5 years in 1567 patients, the power is too small to detect or rule out weak interactions between PRS and treatment. Information on recurrent AF was not continuously collected in both groups and was included as a time-to-event variable in this analysis. A strength is the use of PRS as continuous risk in addition to quintile groups. The grouping of genetic risk by quintiles is largely arbitrary although common practice in genetic studies. No gold standard PRS for AF or stroke is established and different methods for construction were not compared in this study. Our study focuses on a European sample and did not consider ethnicity in the enrolment of the trial. External validity in cohorts of diverse ethnicities is desirable. The choice of anti-arrhythmic drug therapy can be different depending on the country and catheter ablation was only partly used in this study. Therefore, these findings might not generalize to other cohorts of rhythm control. Due to the explorative design of the study, unadjusted *P-*values are given, so descriptive and confidence intervals cannot be used to infer treatment effects. Independent validation is clearly desirable.

## Supplementary material

Supplementary material is available at *Cardiovascular Research* online.

## Supplementary Material

cvad027_Supplementary_Data

## Data Availability

Data will be made available upon request. Please address your proposals for analysis to info@kompetenznetz-vorhofflimmern.de.

## Notes

Translational perspective

- Early rhythm-control therapy reduces cardiovascular outcomes across the spectrum of genetic atrial fibrillation (AF) and stroke risks estimated by polygenic risk scores (PRS).
- The PRS for AF is moderately associated with an increase for recurrent AF while the PRS for stroke is associated with heart failure hospitalization.
- The association of genetic stroke risk with heart failure is replicated in the UK Biobank, highlighting the need for comprehensive care of these cardiovascular conditions.
- Further research into the mechanisms leading to heart failure risk associated with genetic stroke and AF risk could identify approaches for stratified therapy.
