# Supplementary material for: Association of genetic risk and outcomes in patients with atrial fibrillation: interactions with early rhythm control in the EAST-AFNET4 trial
Source: Cardiovasc Res. 2023 Jun 2;119(9):1799–810. doi: 10.1093/cvr/cvad027 (PMC10405565; doi:10.1093/cvr/cvad027)
Supplement: cvad027_Supplementary_Data [file cvad027_supplementary_data.pdf]

SUPPLEMENT:

Polygenic risk scores predict risk of atrial fibrillation recurrence and efficacy of early rhythm control therapy: An EAST-AFNET4 study

Shinwan Kany[1,2,3,4,5*], Christoph Al-Taie[1,2,3*], Carolina Roselli[4], James P. Pirruccello[4,5], Katrin Borof[1,2], Carla Reinbold[1,2], Anna Suling[6], Linda Krause[6], Bruno Reissmann[1,2,3], Renate Schnabel[1,2,3], Tanja Zeller[1,2,3], Antonia Zapf[6], Karl Wegscheider[6], Larissa Fabritz[1,2,3,7], Patrick T. Ellinor[4,5], Paulus Kirchhof[1,2,3,7]

[1]Department of Cardiology, University Heart and Vascular Center Hamburg, University Medical Center Hamburg Eppendorf, Hamburg, Germany
[2]University Center of Cardiovascular Science, University Medical Center Hamburg Eppendorf, Hamburg, Germany
[3]German Center for Cardiovascular Research (DZHK), partner site Hamburg/Kiel/Lübeck, Germany
[4]Cardiovascular Disease Initiative, The Broad Institute of MIT and Harvard, Cambridge, MA, USA
[5]Cardiovascular Research Center, Massachusetts General Hospital, Boston, MA, USA
[6]Institute of Medical Biometry and Epidemiology, University Medical Center Hamburg Eppendorf, Hamburg, Germany
[7]Institute of Cardiovascular Sciences, University of Birmingham, United Kingdom

Contents:

Figures:

- Figure S1: Distribution of polygenic risk among treatment groups and risk categories
- Figure S2: Aalen–Johansen cumulative-incidence curves for the first primary outcome by PRS AF score.
- Figure S3: Aalen–Johansen cumulative-incidence curves for the first primary outcome by PRS Stroke score.
-

Tables:

- Table S1: Baseline characteristics by genetic AF risk category.
- Table S2: Baseline characteristics by genetic stroke risk category.
- Table S3: Treatment effect by genetic AF risk category.
- Table S4: Treatment effect by genetic stroke risk category.
- Table S5: Events per person year (incidence per 100 person years) by genetic AF risk.
- Table S6: Events per person year (incidence per 100 person years) by genetic stroke risk.

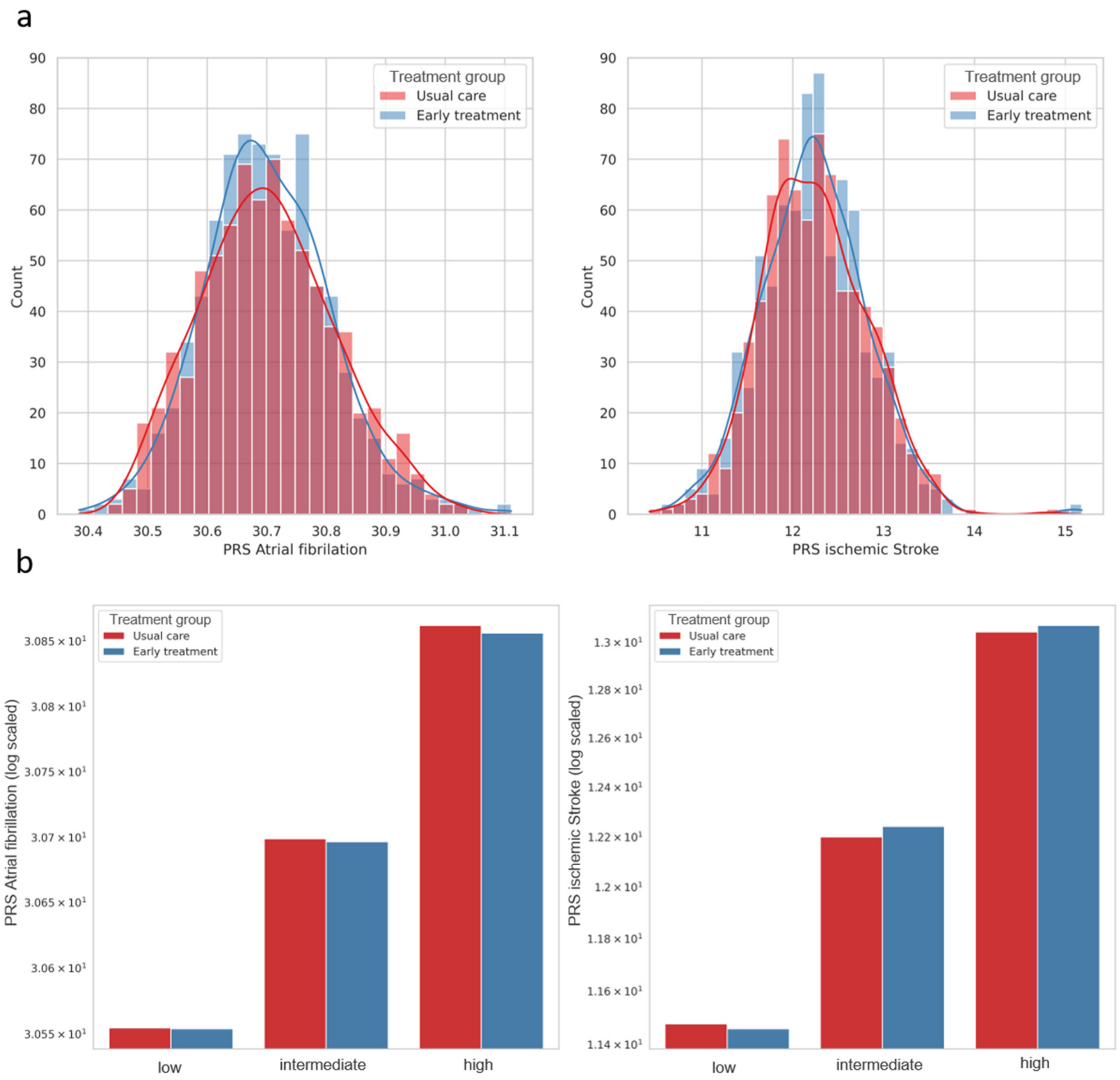

Figure S1: Distribution of polygenic risk scores for a) early rhythm control and usual care and b) grouped by risk categories of low (quintile 5), intermediate (quintile 4-2) and high (quintile 1) risk.

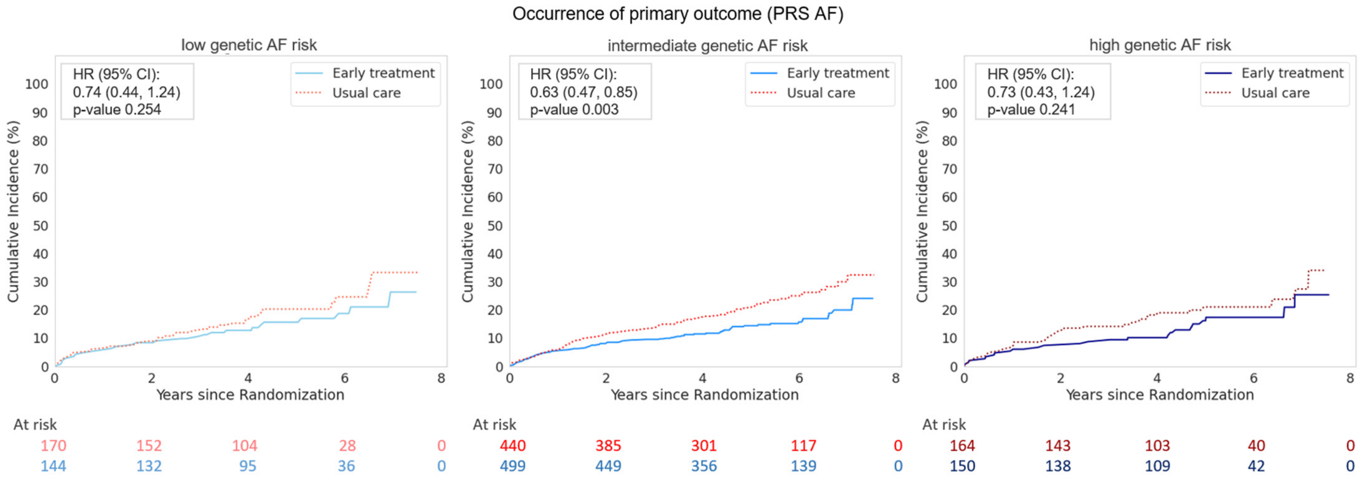

Figure S2: Aalen–Johansen cumulative-incidence curves for the first primary outcome: for patients with low, intermediate and high genetic risk by PRS AF score.

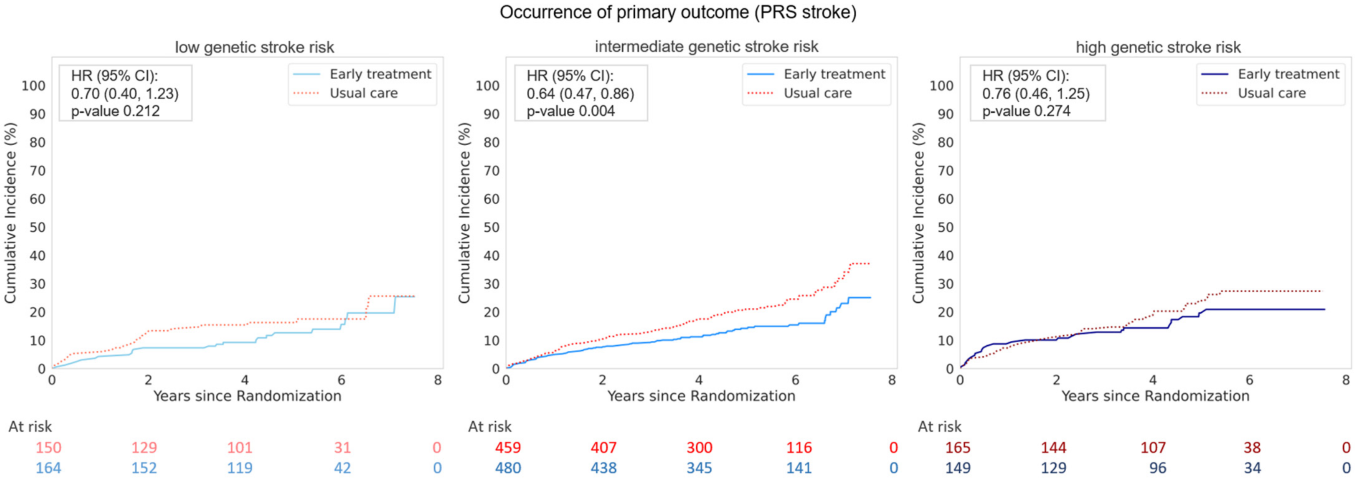

Figure S3: Aalen–Johansen cumulative-incidence curves for the first primary outcome: for patients with low, intermediate and high genetic risk by PRS stroke score.

		PRS AF Score			Total (N=1567)	p-value
		Low (N=314)	Intermediate (N=939)	High (N=314)		
Age	Mean ± SD	71.1 ± 7.8	69.9 ± 8.4	69.2 ± 8.3	70.0 ± 8.3	0.018
	Median [IQR]	72.0 [67.0;76.0]	71.0 [66.0;75.0]	70.0 [65.0;75.0]	71.0 [66.0;76.0]	
Gender (Female)		138 (43.9%)	429 (45.7%)	137 (43.6%)	704 (44.9%)	0.846
Body Mass Index [kg/m²] [N=1560]	Mean ± SD	29.0 ± 5.2	29.5 ± 5.4	29.8 ± 5.2	29.4 ± 5.3	0.252
	Median [IQR]	28.4 [25.2;31.9]	28.7 [25.7;32.3]	29.4 [26.0;33.2]	28.9 [25.6;32.4]	
Type of AF	First episode	118 (37.6%)	344 (36.6%)	93 (29.6%)	555 (35.4%)	0.164
	Paroxysmal	115 (36.6%)	345 (36.7%)	122 (38.9%)	582 (37.1%)	
	Persistent	81 (25.8%)	250 (26.6%)	99 (31.5%)	430 (27.4%)	
Heart rhythm (Sinus rhythm at baseline)		180 (57.3%)	531 (56.5%)	166 (52.9%)	877 (56.0%)	0.837
Days since atrial fibrillation diagnosis	Mean ± SD	74.8 ± 150.6	84.5 ± 199.9	79.0 ± 95.0	81.4 ± 174.0	0.672
	Median [IQR]	37.5 [9.0;99.0]	41.0 [8.0;110.0]	40.5 [9.0;114.0]	40.0 [8.0;107.0]	
Previous cardioversion		130/311 (41.8%)	338/928 (36.4%)	95/310 (30.6%)	563/1549 (36.3%)	0.042
Prior stroke or transient ischemic attack		35 (11.1%)	114 (12.1%)	46 (14.6%)	195 (12.4%)	0.351
**Concomitant conditions**						
At least mild cognitive impairment (MoCA < 26)		131/302 (43.4%)	407/921 (44.2%)	124/303 (40.9%)	662/1526 (43.4%)	0.577
Arterial hypertension		278 (88.5%)	832 (88.6%)	275 (87.6%)	1385 (88.4%)	0.854
Systolic blood pressure [mmHg] [N=1562]	Mean ± SD	137.5 ± 19.7	136.9 ± 19.5	137.3 ± 19.1	137.1 ± 19.4	0.750
	Median [IQR]	135.0 [125.0;145.0]	135.0 [122.0;149.0]	136.0 [125.0;150.0]	135.0 [124.0;149.0]	
Diastolic blood pressure [mmHg] [N=1562]	Mean ± SD	81.3 ± 11.6	81.2 ± 12.0	81.8 ± 12.4	81.4 ± 12.0	0.917
	Median [IQR]	80.0 [73.0;90.0]	80.0 [75.0;90.0]	80.0 [74.0;90.0]	80.0 [74.0;90.0]	
Stable heart failure		90 (28.7%)	265 (28.2%)	116 (36.9%)	471 (30.1%)	0.020
CHA2DS2-Vasc Score	Mean ± SD	3.4 ± 1.3	3.3 ± 1.3	3.3 ± 1.3	3.3 ± 1.3	0.916
	Median [IQR]	3.0 [2.0;4.0]	3.0 [2.0;4.0]	3.0 [2.0;4.0]	3.0 [2.0;4.0]	
Valvular heart disease		130/314 (41.4%)	381/939 (40.6%)	129/313 (41.2%)	640/1566 (40.9%)	0.508
Chronic kidney disease (MDRD stage III or IV)		39 (12.4%)	116 (12.4%)	38 (12.1%)	193 (12.3%)	0.956

Medication at discharge						
Oral anticoagulation (NOAC & VKA) at discharge		292 (93.0%)	861 (91.7%)	287 (91.4%)	1440 (91.9%)	0.823
Digoxin or Digitoxin at discharge		9 (2.9%)	48 (5.1%)	17 (5.4%)	74 (4.7%)	0.349
Beta Blockers at discharge		250 (79.6%)	750 (79.9%)	240 (76.4%)	1240 (79.1%)	0.448
ACE inhibitors or Angiotensin II receptor blocker at discharge		222 (70.7%)	656 (69.9%)	215 (68.5%)	1093 (69.8%)	0.912
Mineralocorticoid receptor antagonist at discharge		17 (5.4%)	66 (7.0%)	18 (5.7%)	101 (6.4%)	0.770
Diuretics at discharge		131 (41.7%)	363 (38.7%)	112 (35.7%)	606 (38.7%)	0.282
Statin at discharge		139 (44.3%)	415 (44.2%)	138 (43.9%)	692 (44.2%)	0.996
Inhibitor of platelet aggregation at discharge		40 (12.7%)	134 (14.3%)	44 (14.0%)	218 (13.9%)	0.868
Rhythm control at baseline	Ablation	11 (3.5%)	30 (3.2%)	15 (4.8%)	56 (3.6%)	0.047
	AAD	133 (42.4%)	469 (49.9%)	133 (42.4%)	735 (46.9%)	
	None	170 (54.1%)	440 (46.9%)	166 (52.9%)	776 (49.5%)	

Note: p-values resulting from mixed linear regression models for metric variables and mixed (ordinal) logistic regression models for categorical variables. For categorical variables with more than two categories (not ordinal) random effect is not included in the model.

Table S1: Baseline characteristics by genetic AF risk category.

		PRS Stroke Score			Total (N=1567)	p-value
		Low (N=314)	Intermediate (N=939)	High (N=314)		
Age	Mean ± SD	70.5 ± 7.9	70.0 ± 8.2	69.6 ± 8.9	70.0 ± 8.3	0.398
	Median [IQR]	71.0 [67.0;76.0]	71.0 [65.0;76.0]	70.0 [65.0;76.0]	71.0 [66.0;76.0]	
Gender (Female)		145 (46.2%)	410 (43.7%)	149 (47.5%)	704 (44.9%)	0.407
Body Mass Index [kg/m^2] [N=1560]	Mean ± SD	29.0 ± 5.2	29.5 ± 5.4	29.6 ± 5.3	29.4 ± 5.3	0.431
	Median [IQR]	28.4 [25.4;32.0]	28.9 [25.8;32.3]	29.1 [25.7;32.9]	28.9 [25.6;32.4]	
Type of AF	First episode	118 (37.6%)	331 (35.3%)	106 (33.8%)	555 (35.4%)	0.897
	Paroxysmal	112 (35.7%)	349 (37.2%)	121 (38.5%)	582 (37.1%)	
	Persistent	84 (26.8%)	259 (27.6%)	87 (27.7%)	430 (27.4%)	
Heart rhythm (Sinus rhythm at baseline)		183 (58.3%)	525 (55.9%)	169 (53.8%)	877 (56.0%)	0.564
Days since atrial fibrillation diagnosis	Mean ± SD	72.9 ± 91.6	84.0 ± 211.9	82.2 ± 92.1	81.4 ± 174.0	0.773
	Median [IQR]	37.0 [6.0;108.0]	38.0 [8.0;103.0]	47.5 [10.0;121.0]	40.0 [8.0;107.0]	
Previous cardioversion		116/308 (37.7%)	345/929 (37.1%)	102/312 (32.7%)	563/1549 (36.3%)	0.164
Prior stroke or transient ischemic attack		39 (12.4%)	108 (11.5%)	48 (15.3%)	195 (12.4%)	0.249
**Concomitant conditions**						
At least mild cognitive impairment (MoCA < 26)		123/306 (40.2%)	393/913 (43.0%)	146/307 (47.6%)	662/1526 (43.4%)	0.185
Arterial hypertension		278 (88.5%)	828 (88.2%)	279 (88.9%)	1385 (88.4%)	0.969
Systolic blood pressure [mmHg] [N=1562]	Mean ± SD	136.7 ± 18.1	137.5 ± 19.9	136.3 ± 19.5	137.1 ± 19.4	0.598
	Median [IQR]	136.0 [125.0;148.0]	136.0 [123.0;150.0]	135.0 [122.0;147.0]	135.0 [124.0;149.0]	
Diastolic blood pressure [mmHg] [N=1562]	Mean ± SD	81.3 ± 11.2	81.6 ± 12.3	80.6 ± 12.0	81.4 ± 12.0	0.622
	Median [IQR]	80.0 [75.0;89.0]	80.0 [74.0;90.0]	80.0 [72.0;89.0]	80.0 [74.0;90.0]	
Stable heart failure		96 (30.6%)	275 (29.3%)	100 (31.8%)	471 (30.1%)	0.563
CHA2DS2-Vasc Score	Mean ± SD	3.4 ± 1.3	3.3 ± 1.3	3.5 ± 1.4	3.3 ± 1.3	0.100
	Median [IQR]	3.0 [2.0;4.0]	3.0 [2.0;4.0]	3.0 [2.0;4.0]	3.0 [2.0;4.0]	
Valvular heart disease		123/313 (39.3%)	390/939 (41.5%)	127/314 (40.4%)	640/1566 (40.9%)	0.615
Chronic kidney disease (MDRD stage III or IV)		36 (11.5%)	111 (11.8%)	46 (14.6%)	193 (12.3%)	0.524

Medication at discharge						
Oral anticoagulation (NOAC & VKA) at discharge		290 (92.4%)	861 (91.7%)	289 (92.0%)	1440 (91.9%)	0.790
Digoxin or Digitoxin at discharge		13 (4.1%)	43 (4.6%)	18 (5.7%)	74 (4.7%)	0.757
Beta Blockers at discharge		246 (78.3%)	733 (78.1%)	261 (83.1%)	1240 (79.1%)	0.203
ACE inhibitors or Angiotensin II receptor blocker at discharge		226 (72.0%)	649 (69.1%)	218 (69.4%)	1093 (69.8%)	0.420
Mineralocorticoid receptor antagonist at discharge		19 (6.1%)	60 (6.4%)	22 (7.0%)	101 (6.4%)	0.926
Diuretics at discharge		133 (42.4%)	345 (36.7%)	128 (40.8%)	606 (38.7%)	0.129
Statin at discharge		138 (43.9%)	404 (43.0%)	150 (47.8%)	692 (44.2%)	0.354
Inhibitor of platelet aggregation at discharge		38 (12.1%)	136 (14.5%)	44 (14.0%)	218 (13.9%)	0.409
Rhythm control at baseline	Ablation	10 (3.2%)	35 (3.7%)	11 (3.5%)	56 (3.6%)	0.459
	AAD	151 (48.1%)	451 (48.0%)	133 (42.4%)	735 (46.9%)	
	None	153 (48.7%)	453 (48.2%)	170 (54.1%)	776 (49.5%)	

Note: p-values resulting from mixed linear regression models for metric variables and mixed (ordinal) logistic regression models for categorical variables. For categorical variables with more than two categories (not ordinal) random effect is not included in the model.

Table S2: Baseline characteristics by genetic stroke risk category.

	Low PRS-AF			Intermediate PRS-AF			High PRS-AF			
	**Early rhythm control**	**Usual care**	**HR (95%-CI)**	**Early rhythm control**	**Usual care**	**HR (95%-CI)**	**Early rhythm control**	**Usual care**	**HR (95%-CI)**	**p interaction**
Primary composite outcome	25/690.0 (3.6)	35/782.0 (4.5)	0.739 (0.439, 1.244)	76/2415.0 (3.1)	101/2156.0 (4.7)	0.629 (0.466, 0.851)	24/705.0 (3.4)	35/791.0 (4.4)	0.729 (0.430, 1.237)	0.806
CV death	5/712.0 (0.7)	14/804.0 (1.7)		24/2533.0 (0.9)	28/2222.0 (1.3)		6/757.0 (0.8)	12/818.0 (1.5)		
Stroke	1/711.0 (0.1)	6/788.0 (0.8)		11/2496.0 (0.4)	19/2176.0 (0.9)		6/748.0 (0.8)	8/805.0 (1.0)		
Worsening of heart failure	19/674.0 (2.8)	15/761.0 (2.0)		42/2447.0 (1.7)	54/2091.0 (2.6)		13/731.0 (1.8)	21/765.0 (2.7)		
Acute coronary syndrome	3/705.0 (0.4)	9/783.0 (1.1)		15/2492.0 (0.6)	24/2166.0 (1.1)		3/744.0 (0.4)	5/802.0 (0.6)		
Recurrent AF	51/505.0 (10.1)	76/506.0 (15.0)		198/1676.0 (11.8)	231/1214.0 (19.0)		73/439.0 (16.6)	84/483.0 (17.4)		

Table S3: Outcomes in the EAST-AFNET4 trial in patients with low, intermediate, and high polygenic risk scores for AF by treatment group. Number of events per person years (incidence per 100 person-years) given. Hazard ratios and p-value resulting from cox proportional hazards model with shared frailty for center and interaction term between random group and PRS AF category. PRS: polygenic risk score; AF: atrial fibrillation; HR: hazard ratio; CI: confidence interval; CV: cardiovascular

	Low PRS-Stroke			Intermediate PRS-Stroke			High PRS-Stroke			
	**Early rhythm control**	**Usual care**	**HR (95%-CI)**	**Early rhythm control**	**Usual care**	**HR (95%-CI)**	**Early rhythm control**	**Usual care**	**HR (95%-CI)**	**p interaction**
Primary composite outcome	24/807.0 (3.0)	27/712.0 (3.8)	0.702 (0.402, 1.226)	74/2319.0 (3.2)	105/2220.0 (4.7)	0.639 (0.474, 0.863)	27/684.0 (3.9)	39/798.0 (4.9)	0.756 (0.457, 1.250)	0.765
CV death	6/835.0 (0.7)	7/740.0 (0.9)		24/2444.0 (1.0)	35/2289.0 (1.5)		5/722.0 (0.7)	12/815.0 (1.5)		
Stroke	5/822.0 (0.6)	5/726.0 (0.7)		9/2425.0 (0.4)	23/2239.0 (1.0)		4/708.0 (0.6)	5/804.0 (0.6)		
Worsening of heart failure	14/816.0 (1.7)	13/704.0 (1.8)		40/2357.0 (1.7)	51/2151.0 (2.4)		20/678.0 (2.9)	26/763.0 (3.4)		
Acute coronary syndrome	4/823.0 (0.5)	9/709.0 (1.3)		14/2410.0 (0.6)	21/2243.0 (0.9)		3/709.0 (0.4)	8/798.0 (1.0)		
Recurrent AF	59/579.0 (10.2)	78/424.0 (18.4)		198/1583.0 (12.5)	227/1326.0 (17.1)		65/457.0 (14.2)	86/454.0 (19.0)		

Table S4: Outcomes in the EAST-AFNET4 trial in patients with low, intermediate, and high polygenic risk scores for ischemic stroke by treatment group. Number of events per person years (incidence per 100 person-years) given. Hazard ratios and p-value resulting from cox proportional hazards model with shared frailty for center and interaction term between random group and PRS stroke category. PRS: polygenic risk score; AF: atrial fibrillation; HR: hazard ratio; CI: confidence interval; CV: cardiovascular

Outcome	Low PRS-AF	Intermediate PRS-AF	High PRS-AF
First primary outcome — events/person-yrs. (incidence/100 person-yrs.)	60/1472.0 (4.1)	177/4570.0 (3.9)	59/1497.0 (3.9)
Components of first primary outcome — events/person-yr (incidence/100 person-yr)			
• Death from cardiovascular causes	19/1516.0 (1.3)	52/4755.0 (1.1)	18/1575.0 (1.1)
• Stroke	7/1499.0 (0.5)	30/4672.0 (0.6)	14/1553.0 (0.9)
• Hospitalization with worsening of heart failure	34/1435.0 (2.4)	96/4538.0 (2.1)	34/1496.0 (2.3)
• Hospitalization with acute coronary syndrome	12/1488.0 (0.8)	39/4658.0 (0.8)	8/1546.0 (0.5)
Recurrent AF	127/1011.0 (12.6)	429/2890.0 (14.8)	157/922.0 (17.0)

Table S5: Events per person year (incidence per 100 person years) by genetic AF risk.

Outcome	Low PRS-Stroke	Intermediate PRS-Stroke	High PRS-Stroke
First primary outcome — events/person-yrs. (incidence/100 person-yrs.)	51/1519.0 (3.4)	179/4539.0 (3.9)	66/1481.0 (4.5)
Components of first primary outcome — events/person-yr (incidence/100 person-yr)			
• Death from cardiovascular causes	13/1576.0 (0.8)	59/4734.0 (1.2)	17/1537.0 (1.1)
• Stroke	10/1548.0 (0.6)	32/4664.0 (0.7)	9/1511.0 (0.6)
• Hospitalization with worsening of heart failure	27/1520.0 (1.8)	91/4508.0 (2.0)	46/1441.0 (3.2)
• Hospitalization with acute coronary syndrome	13/1532.0 (0.8)	35/4653.0 (0.8)	11/1506.0 (0.7)
Recurrent AF	137/1003.0 (13.7)	425/2910.0 (14.6)	151/911.0 (16.6)

Table S6: Events per person year (incidence per 100 person years) by genetic stroke risk.
